# 

**Published:** 1898-10

**Authors:** 


### CONTENTS.

ORIGINAL COMMUNICATIONS—

Tuberculosis of Bones and Joints. By Floyd W. McRae, M.D., Atlanta, Ga...... 505

A Few Brief Observations on the Pathology of the Triple Articulations of the Shoulder. By Thomas H. Manley, M.D., New York.... 511

The Treatment of Impacted Biliary Concretions. By E. B. Jackson, M.D., Houston, Tex.... 520

A Report of Some Surgical Cases. By T. R. Garlington, M.D., Rome, Ga. .... 522

The Danger of Chloroform Anesthesia by Gaslight. By Eugene R. Corson, M.D., Savannah, Ga.... 526

SOCIETY REPORTS—

Richmond Academy of Medicine and Surgery.... 528

CORRESPONDENCE—

The Chicago Academy of Medicine.... 530

Macon Items.... 531

Dr. D. Domingo Madan.... 533

EDITORIAL—

Osteopathy.... 537

Medical Journal Advertisements.... 539

The Anesthetist a Specialty.... 540

NEWS AND NOTES.... 547

BOOK REVIEWS, PAMPHLETS, EXCHANGES—

Book Reviews.... 554

Magazines and Exchanges.... 567

Reprints Received.... 565

SELECTIONS AND ABSTRACTS.... 566

MEDICAL ITEMS—Continued....x, xi, xii, xiii, xiv, xv

ADVERTISERS' INDEX TO ADVERTISEMENTS....xvi

CLASSIFIED INDEX TO ADVERTISEMENTS....xxviii

